# Significant Geo-Social Group Discovery over Location-Based Social Network †

**DOI:** 10.3390/s21134551

**Published:** 2021-07-02

**Authors:** Wei Li, Sisi Zlatanova

**Affiliations:** 1 College of Computer Science and Technology, Harbin Engineering University, Harbin 150001, China; 2 Faculty of Arts, Design and Architecture, School of Built Environment, The University of New South Wales, Sydney, NSW 2052, Australia; s.zlatanova@unsw.edu.au

**Keywords:** geo-spatial analysis, spatial information, location-based service (LBS), location-based social network (LBSN), community detection

## Abstract

Geo-social community detection over location-based social networks combining both location and social factors to generate useful computational results has attracted increasing interest from both industrial and academic communities. In this paper, we formulate a novel community model, termed *geo-social group* (GSG), to enforce both spatial and social factors to generate significant computational patterns and to investigate the problem of community detection over location-based social networks. Specifically, GSG detection aims to extract all group-venue clusters, where users are similar to each other in the same group and they are located in a minimum covering circle (MCC) for which the radius is no greater than a distance threshold γ. Then, we present a GSGD algorithm following a three-step paradigm to enumerate all qualified GSGs in a large network. We propose effective optimization techniques to efficiently enumerate all communities in a network. Furthermore, we extend a significant GSG detection problem to top-*k* geo-social group (T*k*GSG) mining. Rather than extracting all qualified GSGs in a network, T*k*GSG aims to return *k* feasibility groups to guarantee the diversity. We prove the hardness of computing the T*k*GSGs. Nevertheless, we propose the effective greedy approach with a guaranteed approximation ratio of 1−1/e. Extensive empirical studies on real and synthetic networks show the superiority of our algorithm when compared with existing methods and demonstrate the effectiveness of our new community model and the efficiency of our optimization techniques.

## 1. Introduction

Graphs have been widely used to model entities (as vertices) and their relationships (as edges). Community detection and community search over massive networks have been broadly studied [1,2,3,4]. Given a graph G=(V,E), community detection is used to cut vertices *V* into meaningful subgraphs/communities, and the vertices are similar to each other in the same community and dissimilar in different communities. Different from the detection problem, the prerequisite of a community search requires an additional set of query vertices *q*, and the aim is to search for a set of *cohesive subgraphs*, each of which should contain all vertices of *q*.

**Figure 1:**
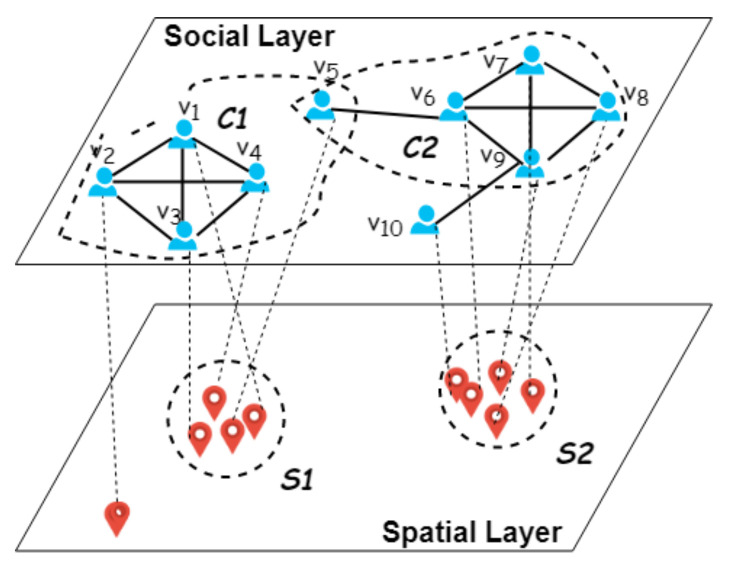
Example of a location-based social network.

As far as the current situation is concerned, most community detection and search methods only focus on the topology of a network. Currently, most of the existing works on community detection and community search only consider the topological structure of a graph. However, with the proliferation of a network along with location such as Twitter, MeetUp, and Foursquare, interest on the topic of community detection/search over a geo-social network has increased [5,6,7,8,9]. In such networks, users are usually associated with location data, which is important for making sense of detected communities (e.g., check-ins and hometown). A typical instance of a location-based social network is shown in Figure 1. Two communities with strong social connections are $C_{1}=\left\{v_{1},v_{2},v_{3},v_{4},v_{5}\right\}$ and $C_{2}=\left\{v_{5},v_{6},v_{7},v_{8},v_{9}\right\}$. Additionally, two communities with strong spatial relationships are $S_{1}=\left\{v_{1},v_{3},v_{4},v_{5}\right\}$ and $S_{2}=\left\{v_{6},v_{7},v_{8},v_{9},v_{10}\right\}$. We use irregular ellipses to represent users’ social relationship and dashed circles to denote users’ physical locations.

Existing Approaches and Limitations. Traditional community detection [1] or subgraph mining approaches [10] mainly focus on link analysis regardless of the spatial features. Some recent works [6,7,8,11] that are based on massive networks associated with physical location information has attracted more and more attention. A spatially oblivious approach to identifying communities is to compute and fuse each pair of vertices’ social distance with its Euclidean distance to define a new geo-social distance (e.g., see [7,8]). Alternatively, different from pairwise geo-social distance, some community search work [6] takes spatial cohesiveness of a community into account, where a community search problem mainly constitutes a query vertices set *q* and a spatial covering threshold. The final output of community search problem in [6] is one community satisfying the following constraints: (1) this community should contain all vertices of *q*; (2) it should satisfy specific structural constraints, and (3) all the community members should be in a *minimum covering circle* with the smallest diameter. However, the problem of a spatial-aware community search requires the inclusion of all given query vertices of *q*, which is inherently different from subgraph mining over location-based social networks.

**Figure 2:**
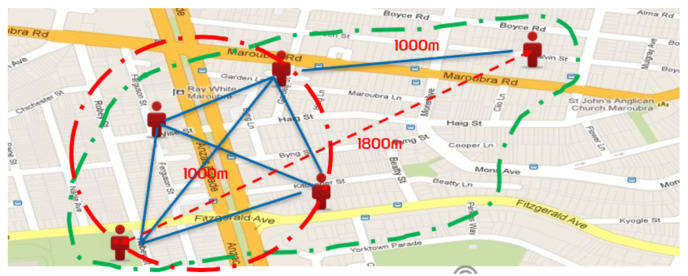
Illustration of the comparison between our model definition and that of Yao et al. [8].

Compute Geo-Social Groups (GSGs). Given a network with location data, in this paper, we aim to detect all qualified subgraphs, called *geo-social groups* (GSGs), where each GSG not only is structurally cohesive but also has strong spatial closeness. This model is more reasonable due to its spatial cohesive definition (see Figure 2, red circle is our detected group and the green one is from Yao et al. [8]). Consider the example in Figure 1: it is obvious that $v_{2}$ is far from the other members in community $C_{1}$. On the contrary, community $C_{1}=\left\{v_{1},v_{3},v_{4},v_{5}\right\}$, as an alternative option, may be more significant for location-based services (LBS), such as event recommendation, social marketing, and geo-social data analysis.

Formally speaking, given a network G=(V,E) and parameters ϵ,μ,γ, where each vertex *v* carries location coordinates (v.x,v.y), a GSG is a set *C* of vertices such that (1) (*structurally cohesive*) the subgraph G[C] of *G* induced by *C* is a structural graph cluster under ϵ and μ and (2) (*spatially cohesive*) vertices in *C* are all within a maximum *minimum covering circle* (MCC) for which the radius is no greater than a distance threshold γ.

Compute Top-*k*Geo-Social Groups (T*k*GSGs). Furthermore, we extend the GSG detection problem to significant top-*k* geo-social group (T*k*GSG) mining. Instead of discovering all location-based group pattern defined in the GSG detection problem, T*k*GSG mining is returns *k* GSGs $C_{k}=\left\{C_{1},...,C_{k}\right\}$ such that (1) all of the *k* GSGs satisfy the social constraints; (2) any GSG $C_{i}\notin C_{k}$ satisfying the social constraints joined in $C_{k}$ results in a union density reduction (see Section 5).

Applications. We now discuss the applications of geo-social group detection.

- **Event recommendation.** Online location-based services, such as Meetup (https://www.meetup.com/ accessed on 4 June 2021), Eventbrite (https://www.eventbrite.com/ accessed on 4 June 2021) and Meetin (https://www.meetin.org/ accessed on 4 June 2021), allow social network users to meet each other physically for entertainment or work purposes (e.g., business forum, dinner, and dating). Suppose that Meetup wishes to recommend different events to users. We can first detect potential groups of users that are spatially and socially close and then recommend events in their vicinity. Intuitively, people who have relatively tight social relations are more likely to participate in a nearby event as a group.
- **Geo-social data analysis.** A common data analysis task is to study features about geographic regions. As discussed in [12], these features are often related to the people located there and their interactions. Another important task is to classify users based on their social connections, tags, geographic locations, and time stamps. GSGD can be employed to provide concrete geo-social context, e.g., by detecting socially dense communities in a geographic area.

Challenges and Our Approaches. In this paper, we address the limitations of previous work as well as propose a novel GSG model that thinks over not only users’ social relationships but also physical distance. To the best of our knowledge, we are the first to formulate and investigate the problem of geo-social group detection and top-*k* GSGs mining over location-based social networks. There are several challenges to tackle.

- First, the GSG detection problem intends to discover a user group within a circle with a radius not larger than γ. How to extract the smallest enclosing circle of a group efficiently is the first challenge.
- Second, it is unclear how to effectively quantify the significance of a set of GSGs, considering that (1) each GSG has a set of vertices as well as an MCC with radius and center, and (2) GSGs may significantly overlap with each other.
- Third, it is also challenging to efficiently compute the set of top-*k* GSGs, considering the large size of real networks as well as the wide variety of radius of MCC.

Contributions. Our contributions are summarized as follows:

- We define a geo-social group model, named GSG, and an effective distance measure among users including both social and physical aspects.
- We formulate the problem of a significant GSG discovery in large geo-social networks and propose effective techniques to generate the candidate set.
- We propose effective pruning techniques to efficiently enumerate the set of all GSG in a network.
- We extend the GSG detection to the significant top-*k* geo-social group (T*k*GSG) discovery problem. Instead of extracting all qualified GSGs in a network, T*k*GSG discovers *k* groups to guarantee the diversity of detection processing while achieving an approximation ratio of 1−1/e.

We conduct extensive empirical studies on large real and synthetic location-based social networks in Section 6. The results show that (i) the formulated community model identifies a set of GSG with both social and spatial cohesiveness guarantee and (ii) our enumerating techniques can can effectively mining all qualified GSGs. Additionally, (iii) our greedy and streaming-like algorithm can achieve high-quality solutions and short run times. Note that the contribution of the significant top-*k* geo-social group (T*k*GSG) discovery listed above is a new extension compared with our conference version in [13].

Organization. The remainder of this paper is structured as follows. Section 2 presents a brief overview of related work; Section 3 defines the geo-social group model and community detection problem. A novel three-step paradigm and efficient GSGD approaches are proposed in Section 4. In Section 5, we present two effective approaches to process the T*k*GSG mining problem. Section 6 reports the experimental results, and Section 7 provides the conclusion and possible future directions.

## 2. Related Work

Besides the above discussed works of spatial-aware community mining over geo-spatial networks, other related works are categorized as follows.

### 2.1. Cohesive Subgraph Extraction

Cohesive subgraph extraction over large networks has been extensively studied, the aim of which is to detect all cohesive subgraphs in a network with a given cohesive definition. Structural graph clustering can be regarded as one way to mine strong structurally cohesive communties, which forms a set of vertices structurally similar to each other and diverse to those who are outside the cluster. In addition to the exact structural graph clustering algorithms discussed above, approximation techniques were studied in [14], where the edge sampling strategy was used to improve the efficiency of an original SCAN algorithm via a reduction in the number of structurally similar computations. Moreover, a cohesiveness definition has unfolded in recent years. For instance, the cohesiveness of a subgraph can be measured by the minimum degree (also known as *k*-core [15,16]), average degree (also known as edge density [17]), minimum number of triangles each edge takes part in (also known as *k*-truss [18,19]), edge connectivity (also known as *k*-edge connected components [20]), etc. These works focus on computing all maximal subgraphs in which the cohesiveness is no less than a user-given threshold. However, these works did not take into account vertices’ location information. Some recent works [21,22] aimed to detect significant subgraphs from spatially constrained networks where vertices in it are labeled with location coordinates [11]. Structural graph clustering [14,23,24] is also a cohesive subgraph regarding given parameters ϵ and μ. However, since inherently different problem definitions and their techniques are inapplicable to our geo-social group mining studied in this paper.

### 2.2. Top-*k* Densest Subgraphs Search

Regarding finding a set of *overlapping* subgraphs over a massive network [25], efficient techniques for finding top-*k* densest subgraphs had been mainly studied.

Computing diversified top-*k* results by considering overlapping among results has also been studied in the literature but for other problems. For example, the problem of computing a set of *k* cliques to cover the most number of vertices is studied in [26], the problem of computing a set of *k* subgraph matchings to cover the most number of vertices is studied in [27], and the problem of computing a set of *k* dense temporal subgraphs to cover the most number of vertex-time instance pairs is studied in [28]. These techniques cannot be applied to compute diversified top-*k* geo-social group (T*k*GSG) mining since our problem definition is inherently different and our diversified score function is also different. Moreover, all of these works have an approximation ratio of 1/4, which is worse than our approximation ratio of 1−1/e.

## 3. Problem Definition

In this paper, we concentrate on an *unweighted, undirected, and location-based network* G(V,E), where *V* is the vertex set and *E* is the set of edges. |V| denotes the number of vertices in *G* by *n*, and |E| denotes the number of edges in *G* by *m*. Let (u,v)∈E represent an edge between two vertices *u* and *v*; *u* (resp. *v*) is considered a neighbor of *v* (resp. *u*). Each vertex *v* in *G* has a location (v.x,v.y), where v.x and v.y are its 2D coordinates along the *x* and *y* axes. In the rest, for ease of presentation, we simply refer to an *unweighted, undirected, and location-based* network as a network.

**Figure 3:**
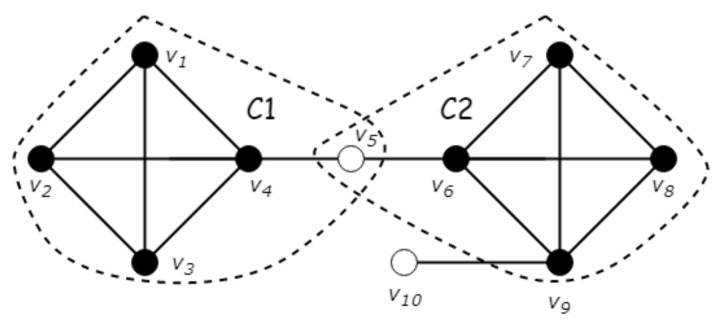
An example graph.

Due to structural graph clustering being adopted for community detection, we refer to the *structural neighborhood* (i.e., *closed neighborhood*) of a vertex *u*, denoted by N(u) (i.e., N(u)={v∈V∣(u,v)∈E}) as the neighbors of *u*. Note that the *open neighborhood* [17] of *u* is N(u)=N[u]∖{u}. The *degree* of vertex *u*, denoted by d[u], is the cardinality of N[u] (i.e., d[u]=|N[u]|). Taking Figure 3 as an example, the *structural neighborhood* of vertex $v_{5}$ is $N\left[v_{5}\right]=\left\{v_{4},v_{5},v_{6}\right\}$, its degree is $d\left[v_{5}\right]=\left|N\left[v_{5}\right]\right|=3$, and its *open neighborhood* is $N\left(v_{5}\right)=\left\{v_{4},v_{6}\right\}$. Considering a vertex subset C⊆V of a graph G(V,E), an induced subgraph of *G* by *C*, denoted by G[C], consists of all vertices in *C* and all edges for which the two endpoints should be in *C* as well (i.e., G[C]=(C,{(u,v)∈E∣u,v∈C})). Table 1 is a summary of the mathematical notations used throughout this paper.

### 3.1. Geo-Social Group (GSG)

Considering a location-based social network G=(V,E), in this paper, our goal is to detect all qualified *geo-social groups*, termed GSGs. Intuitively, a GSG can be regarded as a vertex subset *C* of *V* and subgraph G[C] has a strong cohesiveness value, showing that the group members are closely connected in social and spatial dimensions.

**Structure cohesiveness.** As discussed in the related works in Section 2, several structure cohesiveness measures have been proposed. Thereinto, structural graph clustering [30] can effectively discover hidden structures in a graph. Therefore, we continue to use it in our community structure.

In 2007, Xu et.al. [23] first proposed structural graph clustering, in which the clustering criteria are the neighborhoods of the vertices. They developed the concept of *structural similarity* between vertices *u* and *v*, denoted by σ(u,v), as the number of their common neighbors normalized by the geometric mean of their structural neighborhood sizes. Intuitively, for any two connected vertices, the more common neighbors, the greater the *structural similarity* value. The range of *structural similarity* value is in [0,1]; that is, 0≤σ(u,v)≤1,∀u,v∈V. Given a similarity threshold 0<ϵ≤1 and the minimum number of neighborhoods μ≥2, if σ(u,v)≥ϵ, vertices *u* and *v* can be considered *structurally similar* to each other. When a vertex *u* has no less than μ neighbors that are structurally similar to it, it is a *core vertex*; on the contrary, it is a *non-core vertex*.

If a sequence of vertices $v_{1},v_{2},\ldots ,v_{l}\in V$ (parameter l≥2) in *G* satisfies the following conditions: (1) $v_{1}=u$ and $v_{l}=v$; (2) $v_{1},v_{2},\ldots ,v_{l-1}$ are core vertices; and (3) $v_{i+1}\in N_{\epsilon }\left[v_{i}\right]$ for each 1≤i≤l−1, we say vertex *v* is *structurally reachable* from vertex *u*.

A structurally connected *cluster* *C* is a subset of *V* with no less than two vertices (i.e., |C|≥2) such that we have the following : (i) For any two vertices $v_{1},v_{2}\in C$, there exists a vertex u∈C such that both vertices $v_{1}$ and $v_{2}$ are structurally reachable from *u*; (ii) Once a core vertex u∈C, all vertices that are structurally reachable from *u* are also part of *C*.

**Example** **1.** *Regarding Figure 3, $N\left[v_{5}\right]=\left\{v_{4},v_{5},v_{6}\right\}$ and $N\left[v_{4}\right]=\left\{v_{1},v_{2},v_{3},v_{4},v_{5}\right\}$; thus, $\sigma \left(v_{4},v_{5}\right)=\frac{\left|\{v_{4},v_{5}\}\right|}{\sqrt{3\cdot 5}}=\frac{2}{\sqrt{15}}$. Similarly, $N\left[v_{1}\right]=\left\{v_{1},v_{2},v_{3},v_{4}\right\}$ and $\sigma \left(v_{1},v_{4}\right)=\frac{\left|\{v_{1},v_{2},v_{3},v_{4}\}\right|}{\sqrt{4\cdot 5}}=\frac{2}{\sqrt{5}}$. Given ϵ=0.8 and μ=4, $N_{\epsilon }\left[v_{4}\right]=\left\{v_{1},v_{2},v_{3},v_{4}\right\}$ and $N_{\epsilon }\left[v_{9}\right]=\left\{v_{6},v_{7},v_{8},v_{9},v_{10}\right\}$. Thus, $v_{4}$ and $v_{9}$ are core vertices since $|N_{\epsilon }\left[v_{4}\right]\left|=\right|N_{\epsilon }\left[v_{9}\right]|\geq 4$. Similarly, it is easy to know that $v_{5}$ and $v_{10}$ are non-core vertices. Finally, we obtain two clusters from the graph as shown in Figure 3, $C_{1}=\left\{v_{1},v_{2},v_{3},v_{4},v_{5}\right\}$ and $C_{2}=\left\{v_{5},v_{6},v_{7},v_{8},v_{9}\right\}$.*

**Spatial cohesiveness.** In order to ensure that there is an accessible physical distance among members of the discovered community, a maximum *minimum covering circle* (MCC) is adopted in our community criterion that vertices in a structurally connected *cluster* are required within an MCC in which the radius is no greater than the given user-defined distance threshold γ. Note that the notion of MCC has been broadly adopted to achieve strong spatial closeness for a group of members [6,31,32] in a two-dimensional space. It is defined as follows.

**Definition** **1** (**Minimum Covering Circle** [6])**.** *Given a set of vertices C, the Minimum Covering Circle of C is a spatial circle that can cover all of the vertices in C with the smallest diameter, denoted by MCC(C).*

In this paper, using the structural graph clusters and MCC spatial compactness measure cooperatively, we formally define our GSG community model as follows.

**Definition** **2.** *Given a location-based social network G and three parameters 0<ϵ≤1, μ≥2 and γ>0, a GSG is an induced subgraph* G[C] *in G that satisfies the following constraints.*

- ***Connectivity:*** G[C] *is connected;*
- ***Structure cohesiveness:*** G[C] *is a induced connected subgraph from structural graph clustering, w.r.t. ϵ and μ;*
- ***Spatial cohesiveness:** The MCC of vertices in* G[C] *has radius no larger than γ.*

**Example** **2.** *Given ϵ=0.8 and μ=4, we continue to consider Figure 1. From social layer, two existing groups in social layer are $C_{1}$ = {$v_{1}$,$v_{2}$,$v_{3}$,$v_{4}$,$v_{5}$} and $C_{2}$ = {$v_{5}$,$v_{6}$,$v_{7}$,$v_{8}$,$v_{9}$}. From spatial layer, there are two other communities $S_{1}$ = {$v_{1}$,$v_{3}$,$v_{4}$,$v_{5}$} and $S_{2}$ = {$v_{6},v_{7},v_{8},v_{9},v_{1}0$}. Intuitively, $v_{2}$ is obviously far away from other users in $C_{1}$ so a new community $S_{1}$ = $\left\{v_{1},v_{3},v_{4},v_{5}\right\}$ is more meaningful when considering member positions in actual location based service (LBS). Furthermore, even $v_{10}$ belongs to $S_{2}$; however, it is an outlier for $C_{2}$, new geo-spatial group can be $C_{2}=\left\{v_{6},v_{7},v_{8},v_{9}\right\}$.*

### 3.2. Problem Statement

Along with the aforementioned GSG definitions, we begin to formally define the problem of Geo-Social Group Detection (GSGD) in a network in the following statement:

**Problem Statement.** Given a network G=(V,E) and three parameters 0<ϵ≤1, μ≥2 and γ>0, the problem of GSG detection is to disclose a set of GSG in *G*.

## 4. GSG Detection Algorithm

In this section, we first present our new paradigm in Section 4.1, based on which we then develop our GSGD algorithm that effectively and correctly identifies all GSGs in a massive graph.

### 4.1. Three-Step Paradigm

Our GSG detection algorithm follows a new *three-step paradigm*: (i) clustering core vertices, (ii) clustering non-core vertices, and (iii) computing MCC and discarding invalid clusters.

**Lemma** **1** ([30]). *Each core vertex in the graph must belong to a unique cluster in a structural graph clustering problem.*

**Lemma** **2** ([30]). *Given the clusters of core vertices $C_{c}$ in graph G, non-core vertices in G can be directly clustered by assigning each non-core vertex v to any cluster $C_{c}\in C_{c}$ such that there exists a core vertex $u\in C_{c}$ and $v\in N_{\epsilon }\left[u\right]$.*

Based on Lemmas 1 and 2, our paradigm first computes the clusters of all core vertices by iterating each vertex in graph and by constructing a connected component with only core vertices, and then, the noncore vertices are added to clusters according to Lemma 2.

The minimum covering circle problem (also known as the smallest enclosing circle problem) is a classic computation geometry problem, and most of the geometric approaches look for points that lie on the boundary of the minimum circle and are based on the following lemmas.

**Theorem** **1.** *Given a set of points C, the minimum covering circle (containing all the nodes) is unique.*

**Figure 4:**
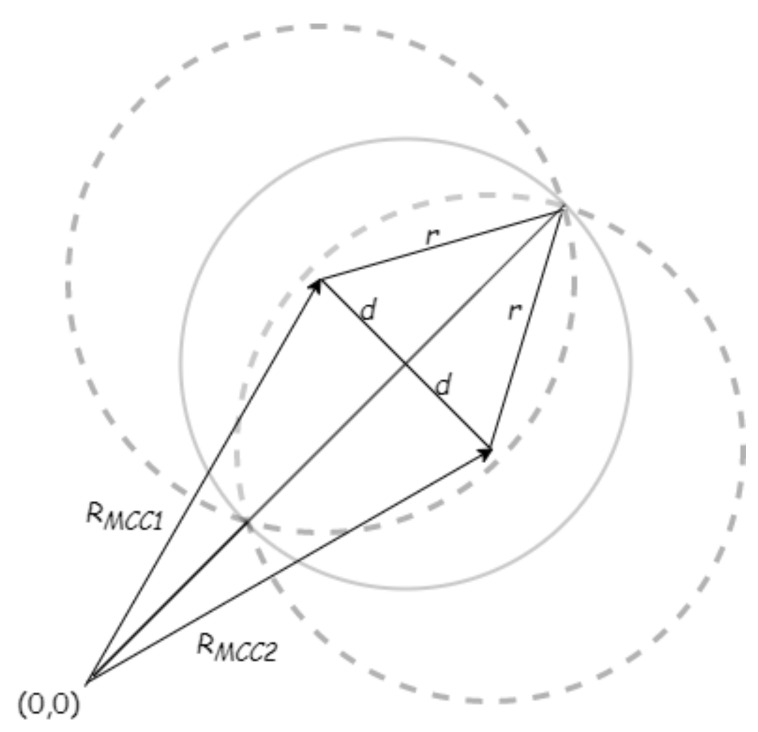
Illustration of the proof of Theorem 1.

**Proof of Theorem** **1.** We prove the theorem by contradiction. Assume that *C* is a set of points in the 2D plane and that ${MCC}_{1}$ and ${MCC}_{2}$ are the centers of two minimum covering circles (MCCs) of set *C*. Then, let *r* be their shared radius and let 2d be the distance between their centers. Due to *C* being a subset of both circles, it must be a subset of their intersection. However, it is easy to see that their intersection is contained within the circles with center $\frac{1}{2}\left({MCC}_{1}+{MCC}_{2}\right)$ and radius $\sqrt{r^{2}-d^{2}}$, as shown in Figure 4. Since *r* is minimal, as a result, $\sqrt{r^{2}-d^{2}}$ must be equal to *r*; that means d=0, so the circles are identical. We reach a contradiction, and the theorem holds. □

**Lemma** **3** ([32]). *Given a vertex set S (|S|≥2), the minimum covering circle (MCC) of S can be determined by up to three vertices in S, which are on the border of the circle. When MCC is determined by only two vertices, the line segment connecting these two vertices must be a diameter of the MCC circle; otherwise, the triangle consisting of three determined vertices is not obtuse.*

Following Lemma 3, it is easy to understand that at least two or three vertices lie on the boundary of the MCC circle of the output GSG.

### 4.2. Our GSGD Approach

Based on the paradigm proposed in Section 4.1, in this section, we present our GSGD approach to geo-social group detection. The pseudocode of our GSGD is shown in Algorithm 1. We initially iterate each pair of adjacent vertices and calculate the structural similarity between them (Line 1–2). In Algorithm 1, we use a disjoint-set data structure [33] to incrementally maintain the connected components when clustering core vertices. The data structure maintains a collection $S=\{S_{1},S_{2},\ldots \}$ of disjoint dynamic subsets and has two fundamental operations: find-subset and union; find-subset determines which subset a particular element is in, and union joins two subsets into a single subset. Line 3 initializes a disjoint-set data structure for each vertex in *G*, and then adding an edge (u,v) into connected component is achieved by union(u,v) (see procedure ClusterCore) (Line 18–19);. Thus, *two vertices u and v are in the same connected component if and only if they are in the same subset in the data structure* (i.e., find-subset(*u*) = find-subset(*v*)). Then, we iteratively scan each vertex *u* in *G* and determine if *u* is a core vertex (Line 3); if so, we unify its neighbors, which are core vertices as well (Line 18–19). The set $C_{c}$ of clusters of core vertices can be formed from the abovementioned disjoint-set data structure (Line 7). After that, clustering of non-core vertices is performed by $C=\{C_{c}{⋃}_{u\in C_{c}}N_{\epsilon }\left[u\right]|C_{c}\in C_{c}\}$ (Line 8). Lastly, we check each cluster *C* in the set C and compute the MCC C of cluster *C* (Line 10–11) when C.r≤γ, *C* is marked as a qualified GSG (Line 9).

**Computing MCC.** The pseudocode for computing the MCC for each cluster derived from Algorithm 1 is given in Algorithms 2 and 3. A naive algorithm (see Algorithm 2) with three nested for-loops take polynomial time $\Theta (n^{4})$. A more efficient randomized incremental method [34] (see Algorithm 3) is adopted in our paper and runs at an expected linear time Θ(n).

**Definition** **3.** *(MCC Center). Center center(x,y) is the position that is at the minimum distance from all vertices on or within the circle.*

**Definition** **4.** *(MCC Radius). Radius r is the radius of the minimum covering circle of a set of points.*

**Algorithm 1:** GSGD

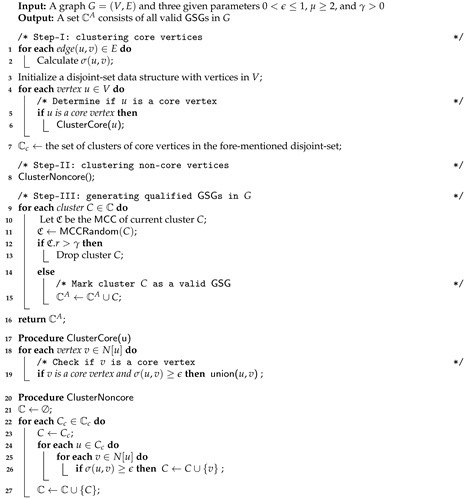

**Example** **3.** *Considering our example in Figure 1 given parameters ϵ=0.8 and μ=4. We first determine core nodes by calculating the structural similarity of each pair of connected nodes, e.g., $\sigma \left(v_{1},v_{2}\right)=\sigma \left(v_{1},v_{3}\right)=\sigma \left(v_{2},v_{3}\right)=\sigma \left(v_{7},v_{8}\right)=\sigma \left(v_{8},v_{9}\right)=\sigma \left(v_{7},v_{9}\right)=1$, $\sigma \left(v_{1},v_{4}\right)=\sigma \left(v_{2},v_{4}\right)=\sigma \left(v_{3},v_{4}\right)=\sigma \left(v_{6},v_{7}\right)=\sigma \left(v_{6},v_{9}\right)=\sigma \left(v_{6},v_{8}\right)=\frac{2}{\sqrt{5}}$, and $\sigma \left(v_{4},v_{5}\right)=\sigma \left(v_{5},v_{6}\right)=\frac{2}{\sqrt{15}}$. After that, we iterate each node in the graph and calculate $N_{\epsilon }\left[v_{4}\right]=\left\{v_{1},v_{2},v_{3},v_{4}\right\}$ and $N_{\epsilon }\left[v_{9}\right]=\left\{v_{6},v_{7},v_{8},v_{9},v_{1}0\right\}$. Thus, $v_{4}$ and $v_{9}$ are two unique core vertices since $|N_{\epsilon }\left[v_{4}\right]\left|=\right|N_{\epsilon }\left[v_{9}\right]|\geq 4$. In Algorithm 1, non-core vertices (e.g., $v_{1},v_{2},v_{3},v_{5},...$) are directly assigned to corresponding core clusters by Lemma 2; that is, vertices $v_{1},v_{2},v_{3},v_{5}$ belonging to cluster $C_{1}$ and $v_{5},v_{6},v_{7},v_{8}$ are in cluster $C_{2}$. Next, we traverse clusters $C_{1}$ and $C_{2}$ and compute MCC of current cluster through Algorithm 3. We know that $v_{2}$ is eliminated from $C_{1}$ and that $v_{5}$ does not belong to $C_{2}$ as well. Finally, two valid GSGs are $C_{1}=\left\{v_{1},v_{3},v_{4},v_{5}\right\}$ and $C_{2}=\left\{v_{6},v_{7},v_{8},v_{9}\right\}$.*

**Algorithm 2:** MCCNaive(*C*)

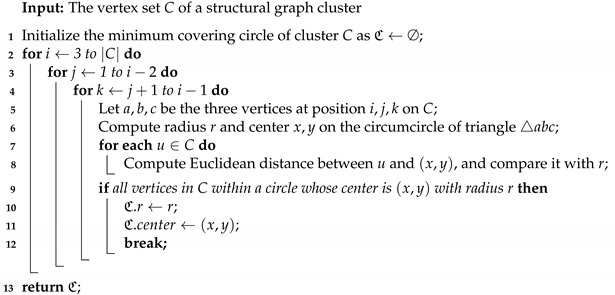



### 4.3. Optimization Techniques

In this section, we propose optimization techniques to improve the efficiency of GSGD.

**(1) Efficiently Compute Structural Similarity between Two Vertices.** GSGD (i.e., Algorithm 1) computes σ(u,v) when exploring *u* and σ(v,u) when exploring *v*. These two structurally similar calculations significantly increase computational burden. Therefore, we adopt the cross link technique, which links edge (u,v) with edge (v,u); then, the structural similarity between *u* and *v* only needs to be calculated once. Overall, the size of structural similarity calculations in a graph is expected to be reduced by half. Concerning time complexity, we assume that the adjacent list of each vertex *u* is ordered by vertex IDs; then, we can utilize a binary search (with time complexity of O(logd[v])) on N[v] to search edge (v,u) when (u,v) is processed. In total, the time complexity of this cross link is $O(\sum_{(u,v)\in E}\log\left\{\min\left\{d\left[u\right],d\left[v\right]\right\}\right\})$.

**(2) Spatial-Structural Neighborhood Pruning Rules.** Due to spatial cohesiveness constraints, we consider the physical distance between vertices when computing clustering in Phase-I. Thus, rather than focusing on pure structural neighborhood, we redefine the neighborhood of a vertex *u* in *G* by considering the physical distance between *u* and N[u], as follows.

**Definition** **5.** *(Spatial-Structural Neighborhood). The **spatial-structural neighborhood** of a vertex u, denoted by $N_{\gamma }\left[u\right]$, is defined as a set of vertices in the structural neighborhood of u, and the distance between them and vertex u is not greater than γ; that is, $N_{\gamma }\left[u\right]=\left\{v\in V|\left(u,v\right)\in E\wedge dist\left(u,v\right)\leq \gamma \right\}\cup \left\{u\right\}$, where dist(u,v) is the spatial distance between u and v.*

Intuitively, two vertices *u* and *v* cannot be in the same geo-social group if the distance between them is already larger than the spatial threshold γ according to the definition of GSG.

**Algorithm 3:** MCCRandom(*C*)

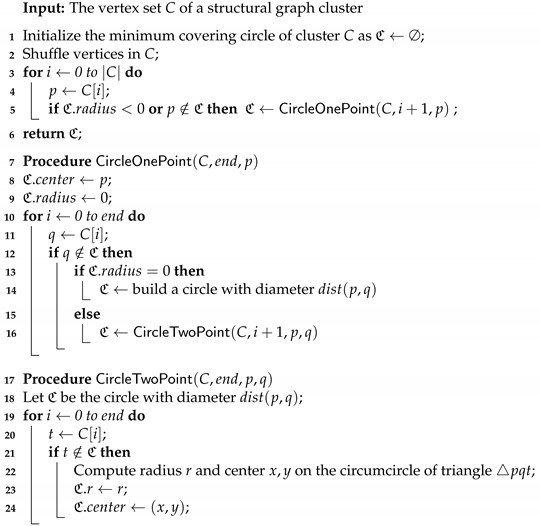



## 5. Top-*k* Densest GSGs

In this section, we present our basic definitions and formulate the top-*k* densest GSG search problem. Instead of finding all valid geo-social group defined in the GSG detection, the top-*k* densest GSGs search returns a set of *k* GSGs $C_{k}^{*}=\left\{C_{1}^{*},C_{2}^{*},...,C_{k}^{*}\right\}$.

**Density of a GSG.** Intuitively, the larger the size |C| of the GSG and the smaller the radius *r* of MCC, the more important the GSG. Thus, we define a density of a GSG as $\rho \left(C\right)=\frac{\left|C\right|}{r}$. For example, consider Example 3. The GSG $C_{1}=\left\{v_{1},...,v_{5}\right\}$ has five vertices, and the radius is 20; thus, $\rho \left(C_{1}\right)=\frac{5}{20}=0.4$. Formally, we define the density as follows.

**Definition** **6.** *(Density). Given a geo-social group GSG C and its MCC radius r, the density of such a GSG C is defined as $\rho \left(C\right)=\frac{\left|C\right|}{r}$.*

**Definition** **7.** (Densest GSG). Given a geo-social graph G=(V,E), the densest GSG $C^{*}$ is a GSG that maximizes the density function, i.e., $C^{*}={\arg\max}_{C\subseteq V}\rho (C)$.

**Diversified Density of a Set of GSGs.** Given a set C of GSGs, a naive approach to quantifying the score of C is to sum up ρ(C) for each GSG *C* in C. However, this may significantly overestimate the score of C in view of the overlaps that may exist among the GSG. Based on the observations discussed in [30], the resulting clusters may overlap.

In view of the above, we first define the diversified union density of a set C of GSG, denoted ρ(C) as follows.

**Definition** **8.** *(Diversified Union Density). The diversified union density of a set C of GSGs is defined as $\rho \left(C\right)=\sum_{u\in C}\max_{C\in C,(u,r)\in C}\frac{1}{r}$.*

The rationality of our definition is that, although a vertex *u* may be covered by many GSG of C, we only discount its weight based on the radius of the smallest GSG that covers *u*. Note that the union discussed in this paper uses the set-based semantics; that is, each element is included into the union result at most once.

### 5.1. Problem Statement

Armed with the above definitions, we are ready to define our problem of top-*k* GSG selection as follows.

**Problem Statement.** Given a geo-social network G=(V,E) and parameters 0<ϵ≤1, μ≥2, and *k*, the problem of the top-*k* densest GSG selection is to uncover a set of *k* GSGs $C_{k}^{*}=\left\{C_{1}^{*},C_{2}^{*},...,C_{k}^{*}\right\}$ for which the diversified union density is the largest among all sets of *k* GSGs in *G*.

**Hardness Analysis.** Assuming that the set of all GSGs in *G* has been generated and stored in $C^{A}$, it it still a hard problem to extract the set of top-*k* GSGs from $C^{A}$. Intuitively, given $C^{A}$, our problem becomes computing the set of *k* GSGs from $C^{A}$ that covers the most vertices with the smallest MCC radius, which is an instance of the NP-hard *k*-set-coverage problem [35]. As a result, to exactly compute the set of top-*k* GSGs from $C^{A}$, we may need to enumerate all sets of *k* GSGs of $C^{A}$, to compute their density, and to finally return the set of *k* GSGs with the maximum union density. Moreover, it is worth noting that $C^{A}$ can be of exponential size with respect to the size of *G* in the worst case.

### 5.2. A Greedy Approach

According to the hardness of computing the optimal GSG exactly or approximately, we propose a two-phase approach to computing top-*k* GSGs by (*Phase-I*) first exhaustively generating the set $C^{A}$ of all GSGs and (*Phase-II*) by then selecting the top-*k* ones from $C^{A}$.

**Phase-I: Generate All GSGs $C^{A}$.** As we have demonstrated how to enumerate a qualified MCC under the condition that the radius of GSG’s MCC should be less than a threshold in Section 4, we can release the above radius condition and regard each cluster as a valid GSG with an MCC which covers all vertices in that GSG.

Based on this revision, the pseudocode generating all GSGs in a graph is shown in Algorithm 4. We compute the structural similarities for all pairs of adjacent vertices (Line 1). In Lines 2–6, we continue to cluster core vertices, and then, Line 7 clusters non-core vertices. After that, MCC can be calculated using Algorithm 3 for each cluster in the graph. Instead of dropping all clusters for which the MCC radius is less than the defined threshold, we keep all generated clusters and its MCC radius in order to select the following top-*k* GSGs in *Phase-II*.

**Phase-II: Greedily Select Top-*k*GSGs.** As discussed in Section 5, given $C^{A}$, it is still a hard problem to select the top-*k* GSGs from $C^{A}$. The good news is that our density of a set of GSGs (see Definition 8) is monotone and submodular, as proven in the lemma below.

**Lemma** **4.** *For any two sets of GSGs, $C_{1}$ and $C_{2}$, such that $C_{1}\subseteq C_{2}$, we have $\rho \left(C_{1}\right)\leq \rho \left(C_{2}\right)$ (**Monotone**). Moreover, for any GSG $C\notin C_{2}$, we have $\rho \left(C_{1}\cup \left\{C\right\}\right)-\rho \left(C_{1}\right)\geq \rho \left(C_{2}\cup \left\{C\right\}\right)-\rho \left(C_{2}\right)$ (**Submodular**).*

**Proof of Lemma** **4.** Due to only discounting the weight of vertex *u* based on the size of the smallest GSG that covers *u*, we use $\omega_{C}\left(u\right)$ to denote the weight of *u* in C, which is $\max_{C\in C,u\in C}\frac{1}{\left|C\right|}$. Note that (1) $\omega_{C}\left(u\right)=0$ if u∉C and (2) $\omega_{C}\left(u\right)=\max\left\{\omega_{C_{1}}\left(u\right),\omega_{C\setminus C_{1}}\left(u\right)\right\}$ for any $C_{1}⊊C$.

**Figure 5:**
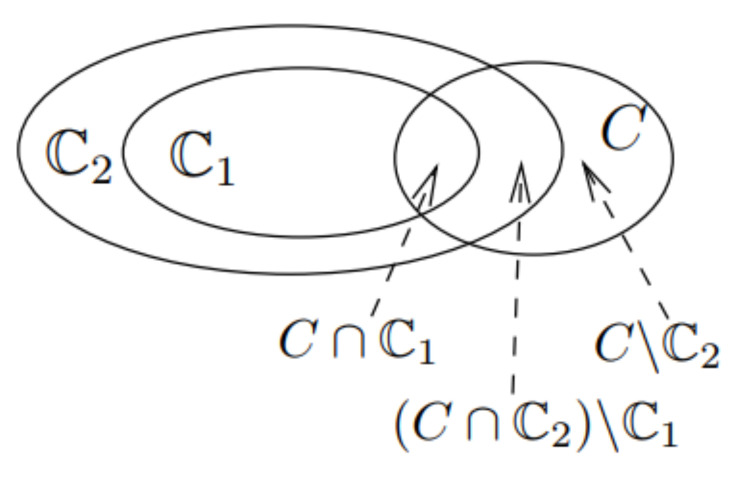
Illustration of the proof of Lemma 4.

As every vertex covered by $C_{1}$ is also covered by $C_{2}$, we have $\rho \left(C_{1}\right)\subseteq \rho \left(C_{2}\right)$ as illustrated in Figure 5, where each ellipse represents the set of vertices that are covered by the GSG or the set of GSGs. Moreover, for each $u\in C_{1}$, we have $\omega_{C_{1}}\left(u\right)\leq \omega_{C_{2}}\left(u\right)$ since $C_{1}\subseteq C_{2}$. Thus, $\rho \left(C_{1}\right)\leq \rho \left(C_{2}\right)$ holds.

**Algorithm 4:** Greedy

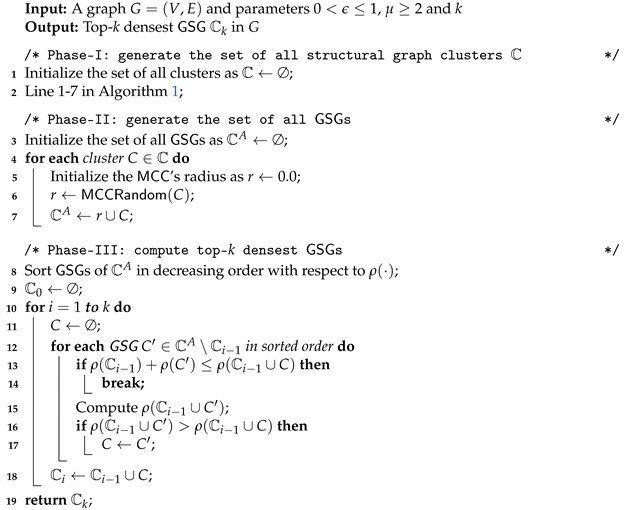



Now, we prove the submodularity of ρ(·). According to the definition, we have $\rho \left(C_{1}\cup \left\{C\right\}\right)-\rho \left(C_{1}\right)=\sum_{u\in C_{1}\cup C}\left(\omega_{C_{1}\cup \left\{C\right\}}\left(u\right)-\omega_{C_{1}}\left(u\right)\right)$. Note that (1) $\omega_{C_{1}\cup \left\{C\right\}}\left(u\right)=\omega_{C_{1}}\left(u\right)$ for each $u\in C_{1}\setminus C$ and (2) we can partition *C* into three parts, $C\cap C_{1}$, $\left(C\cap C_{2}\right)\setminus C_{1}$, and $C\setminus C_{2}$, as shown in Figure 5. Thus, we have $\rho \left(C_{1}\cup \left\{C\right\}\right)-\rho \left(C_{1}\right)=\sum_{u\in C\cap C_{1}}\left(\omega_{C_{1}\cup \left\{C\right\}}\left(u\right)-\omega_{C_{1}}\left(u\right)\right)+\sum_{u\in \left(C\cap C_{2}\right)\setminus C_{1}}\frac{1}{\left|C\right|}+\sum_{u\in C\setminus C_{2}}\frac{1}{\left|C\right|}$. Now, we compare the three components of $\rho \left(C_{1}\cup \left\{C\right\}\right)-\rho \left(C_{1}\right)$ with that of $\rho \left(C_{2}\cup \left\{C\right\}\right)-\rho \left(C_{2}\right)$ one by one. First, for every $u\in \left(C\cap C_{2}\right)\setminus C_{1}$, we have $\omega_{C_{2}\cup \left\{C\right\}}\left(u\right)-\omega_{C_{2}}\left(u\right)\leq \frac{1}{\left|C\right|}$, since $\omega_{C_{2}\cup \left\{C\right\}}\left(u\right)=\max\left\{\frac{1}{\left|C\right|},\omega_{C_{2}}\left(u\right)\right\}$. Second, for every $u\in C\cap C_{1}$, we consider $\left(\omega_{C_{1}\cup \left\{C\right\}}\left(u\right)-\omega_{C_{1}}\left(u\right)\right)-\left(\omega_{C_{2}\cup \left\{C\right\}}\left(u\right)-\omega_{C_{2}}\left(u\right)\right)$ in two cases. If $\omega_{C_{2}\cup \left\{C\right\}}\left(u\right)=\omega_{C_{2}}\left(u\right)$, we have $\omega_{C_{1}\cup \left\{C\right\}}\left(u\right)\geq \omega_{C_{1}}\left(u\right)$; otherwise, $\omega_{C_{2}\cup \left\{C\right\}}\left(u\right)=\frac{1}{\left|C\right|}$, and we have $\omega_{C_{1}\cup \left\{C\right\}}\left(u\right)=\frac{1}{\left|C\right|}$ and $\omega_{C_{2}}\left(u\right)\geq \omega_{C_{1}}\left(u\right)$, since $C_{1}\subseteq C_{2}$. As a result, we have $\rho \left(C_{1}\cup \left\{C\right\}\right)-\rho \left(C_{1}\right)\geq \rho \left(C_{2}\cup \left\{C\right\}\right)-\rho \left(C_{2}\right)$. □

Following Lemma 4, a greedy algorithm can be designed to compute a result with an approximation ratio of 1−1/e, as follows. Given an initially empty C, we iteratively add into C the GSG *C* that together with C result in the largest total density; that is, $C=\arg\max_{C^{\prime }\in C^{A}\setminus C}\rho \left(C\cup \left\{C^{\prime }\right\}\right)$. The pseudocode of the greedy selection is shown at Lines 14–16 of Algorithm 4. Thus, Algorithm 4 reports the exact top-*k* GSG when k=1, and the general approximation ratio of Algorithm 4 is proven by the theorem below.

**Theorem** **2.** Given a network G and parameters k,γ,ϵ,μ, let $C_{k}^{*}$ be the optimal top-k GSGs and $C_{k}$ be the set of k GSGs obtained by Algorithm 4. Then, we have $\rho \left(C_{k}\right)\geq \left(1-1/e\right)\times \rho \left(C_{k}^{*}\right)$,

**Proof of Theorem ** **2.** This theorem directly follows from Lemma 4 and the results in [36]. □

### 5.3. A Swap-Based Approach

Greedy needs to store the set $C^{A}$ of all GSGs, which may be, in the worst case, too large to be stored in the main memory. If this is the case, then we can switch our algorithm to a stream-like processing in a similar fashion to the existing works on diversified top-*k* search (e.g., [27]). That is, we maintain a set $C_{k}$ of at most *k* GSGs during the execution of the algorithm. After obtaining a new GSG *C* (e.g., at Line 14 of Algorithm 4), rather than storing it in $C^{A}$, we directly update $C_{k}$ by *C*. Specifically, if $C_{k}$ contains less than *k* GSGs, then we insert *C* into $C_{k}$. Otherwise, we try to replace one of the GSG in $C_{k}$ with *C*, as follows. Let $C_{k+1}=C_{k}\cup \left\{C\right\}$, and let $C^{\prime }=\arg\max_{C^{\prime \prime }\in C_{k+1}}\rho \left(C_{k+1}\setminus \left\{C^{\prime \prime }\right\}\right)$. Then, we insert *C* into $C_{k}$ and remove $C^{\prime }$ from $C_{k}$ if $\rho \left(C_{k+1}\setminus \left\{C^{\prime }\right\}\right)\geq \left(1+\frac{1}{k}\right)\times \rho \left(C_{k}\right)$, and keep $C_{k}$ unchanged otherwise. We denote this algorithm by Swap as shown in Algorithm 5.

**Algorithm 5:** Swap

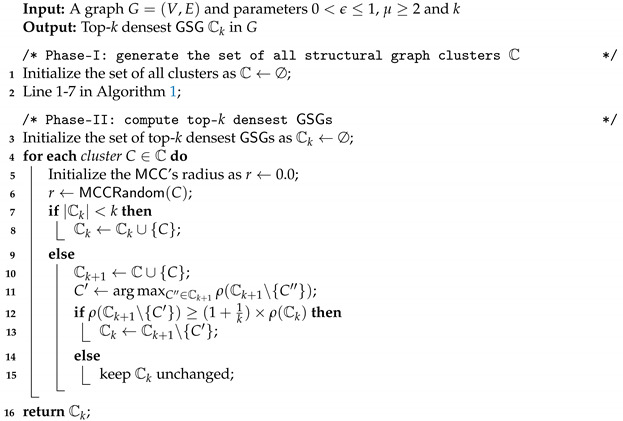



### 5.4. Optimization Techniques

**Efficiently Select Top-*k*GSGs.** In Algorithm 4, given a subset C of $C^{A}$, we need to select the next GSG *C* such that ρ(C∪{C}) is maximized. One naive approach is computing $\rho (C\cup \left\{C^{\prime }\right\})$ for every GSG $C^{\prime }\in C^{A}\setminus C$, and then selecting the best one. However, this is time-consuming if $|C^{A}|$ becomes large. Thus, we develop an upper bound-based pruning as follows.

Note that $\rho \left(C\cup \left\{C^{\prime }\right\}\right)\leq \rho \left(C\right)+\rho \left(C^{\prime }\right)$; thus, $\rho \left(C\right)+\rho \left(C^{\prime }\right)$ can be regarded as an upper bound of $\rho (C\cup \left\{C^{\prime }\right\})$. We first sort all GSGs of $C^{A}$ in decreasing order with respect to their individual ρ(·) scores, which is also the decreasing order with respect to their upper bounds. Thus, we process GSGs in $C^{A}$ in decreasing order with respect to upper bounds and terminate them once the upper bound becomes not larger than ρ(C∪{C}), where *C* is the currently best selected one. As a result, we do not need to process the entire list of GSGs in $C^{A}$ each time.

## 6. Experiments

We first provide a description of the experiment setup in Section 6.1. Section 6.2 and Section 6.3 report the effectiveness and efficiency evaluations of our GSG detection and top-*k* densest GSGs mining approaches. The source code is hosted on GitHub (https://github.com/weil0819/gsgd accessed on 4 June 2021).

### 6.1. Setup

First, we evaluate the efficiency and effectiveness of our proposed approaches for geo-social groups (GSGs) detection and compare it with the-state-of-the art method DGCD [8].

- Naive: the geo-social group detection algorithm with naive approach for MCC in Section 4.
- Random: the geo-social group detection algorithm with randomized incremental construction for MCC in Section 4.
- DGCD: the state-of-the-art density-based geo-community detection algorithm in [8].

Secondly, we evaluate the following proposed algorithms for Top-*k* densest geo-social groups (T*k*GSGs) mining.

- Greedy: our greedy approach for top-*k* densest geo-social group mining in Section 5.
- Swap: our swap approach for top-*k* densest geo-social group mining in Section 5.

All algorithms in this paper are implemented in C++ (single thread) and complied by GNU GCC 4.8.2 with the –O3 optimization. All experiments in this paper are conducted on a machine with an Intel(R) Core(TM) i5-6200U 2.3 GHz CPU and 8 GB main memory running 64-bit Ubuntu Linux.

**Datasets.** We evaluate the performance of all algorithms on two real networks and three synthetic networks as summarized in Table 2, among which Brightkite and Gowalla have real location labels. Note that we only consider the first check-in position as the user’s geographic location. We also evaluate the proposed methods on LFR benchmark networks [37] by increasing the number of vertices from $10^{4}$ to $10^{6}$, and the default average clustering coefficient is set to 0.2; this value is derived from the Brightkite and Gowalla datasets. Furthermore, akin to [30], we fix the average and maximum degree of the whole network at 20 and 50, respectively. For each synthetic graph, we generate node position in a similar manner to [6,8], as follows. First, we randomly pick a node *v* and assign it a random position in the space [0,1000]×[0,1000]. Then, following the normal distribution with mean 300 and standard deviation 600, we put *v*’s neighbors at random positions. Starting from *v*’s neighbors, we repeat the above two steps until every node in the graph has a location.

**Parameters.** For each experimental network, we vary the three parameters in Section 6.2 and Section 6.3: 0<ϵ≤1, μ≥2, and γ>0. Concerning ϵ, we select 0.4, 0.5, 0.6, and 0.7, with ϵ=0.6 as the default. For μ, we choose 5, 10, 15, and 20, with μ=10 as the default. For γ, we choose 25, 50, 75, and 100, with γ=50 as the default.

**Metrics.** We evaluate the algorithms from two aspects: effectiveness and efficiency. Regarding effectiveness, we evaluate the total number of GSGs and our three-step paradigm. Regarding efficiency, we evaluate the total processing time by running an algorithm three times and by reporting the average CPU time.

### 6.2. Performance of GSG Detection

**Eval-I: Total Number of GSGs.** As both of our algorithms GSGD with NaiveMCC (Naive for short), GSGD with RandomMCC (Random for short) need to generate and keep all candidate GSGs and then calculate MCC for each candidate, **Eval-I** first evaluates the total number of GSGs in a network. Given μ=5 and γ=50, as both Naive and Random generate the same $C^{A}$, we just need to run one of them. The result under different ϵ values are shown in Table 3. It is easy to understand that the number of GSGs decreases when ϵ becomes larger. We can see that, the larger ϵ, the less possible communities. Nevertheless, it is still manageable even for ϵ=0.4. Thus, our strategy of storing all candidate GSGs works in practice.

**Figure 6:**
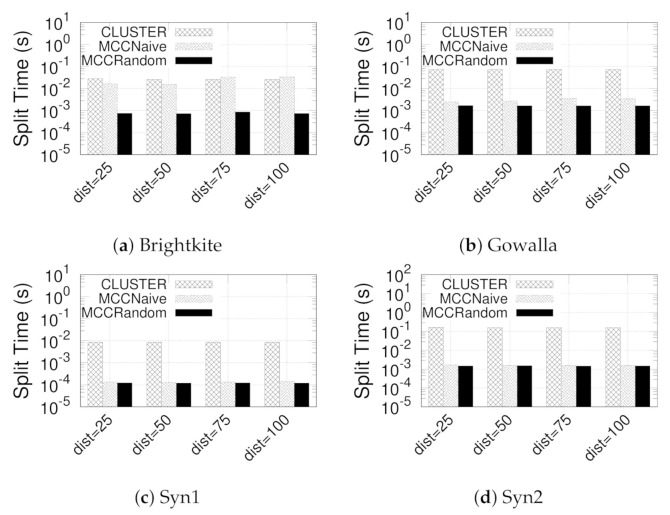
(Eval-II) Split time of clustering and MCC computation.

**Eval-II: Evaluating Our Three-step Paradigm.** We evaluate the efficiency of designed three-step paradigm by comparing the time of clustering and MCC computation. Recall from Section 4.1 and Section 4.2 that our proposed paradigm consists of three steps: (Step-I) clustering core nodes, (Step-II) clustering non-core nodes, and (Step-III) computing MCC and discarding invalid subgraphs. We pack Step-I and Step-II together as CLUSTER and Step-III individual as MCCNaive and MCCRandom, respectively. The processing times of CLUSTER, MCCNaive, and MCCRandom are presented in Figure 6 by varying γ. The processing time of CLUSTER remains almost the same when γ increases from 25 to 100 because Step-I, which is irrelevant to γ, is the dominating cost of GSGs detection. On the other hand, the run time of MCCNaive and MCCRandom increases slightly when γ increases; this is due to the search range of Step-II, which increases with γ. Nevertheless, MCCRandom consistently performs better than MCCNaive; this is due to the linear time complexity.

**Figure 7:**
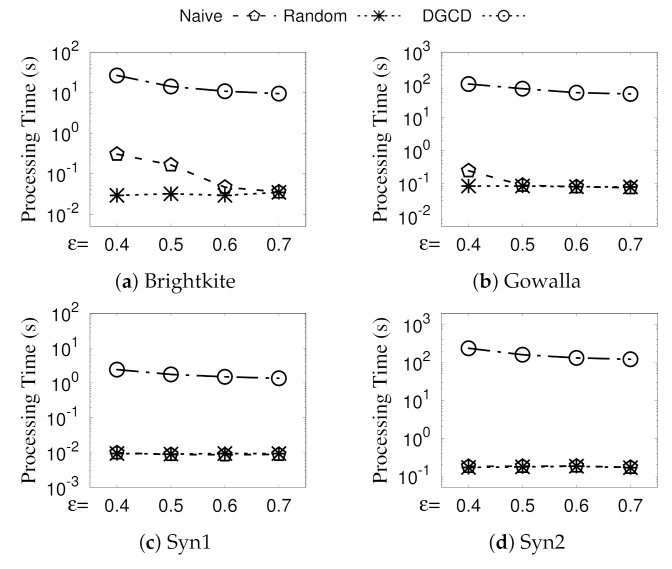
(Eval-III) Against existing algorithms (vary ϵ).

**Eval-III: Vary ϵ.** The run time of GSGD with NaiveMCC (Naive for short), GSGD with RandomMCC (Random for short), and DGCD by varying ϵ is illustrated in Figure 7. The processing time of Random is kept steady for different ϵ because of its linear time complexity. When ϵ increases, it is more likely that two adjacent vertices are not structurally similar to each other and, thus, can be prepruned. Note that, for a larger ϵ, DGCD runs on less time. The reason for this is that a vertex is less likely to be a core vertex for a larger ϵ. In summary, GSGD is significantly faster than DGCD by more than two orders of magnitude for all ϵ.

**Figure 8:**
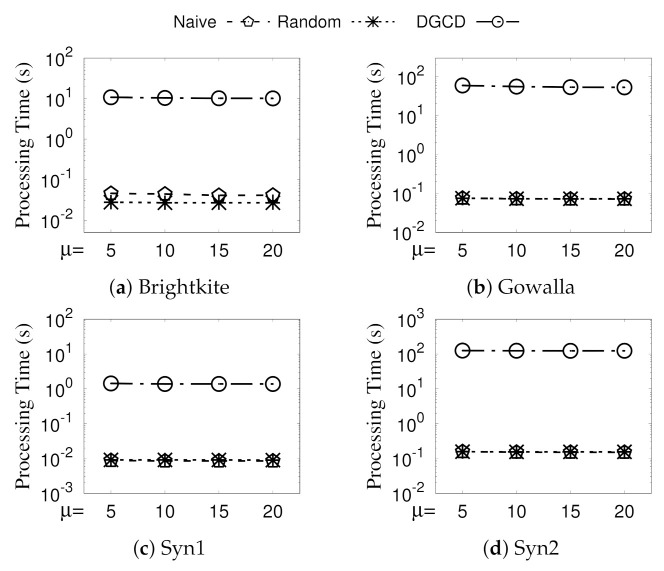
(Eval-IV) Against existing algorithms (vary μ).

**Eval-IV: Vary**μ. Figure 8 presents the performances of GSGD (with NaiveMCC and RandomMCC) and DGCD by varying μ. Basically, the processing times of both GSGD and DGCD hold steady for different μ values. GSGD takes slightly less time when μ increases. That is because, as μ increase, more vertices can be pruned to be core vertices (see Section 4.1) in our paradigm; thus, less structurally similar computations are found between core vertices along with less time cost. Furthermore, GSGD consistently outperforms DGCD regarding parameter μ.

**Figure 9:**
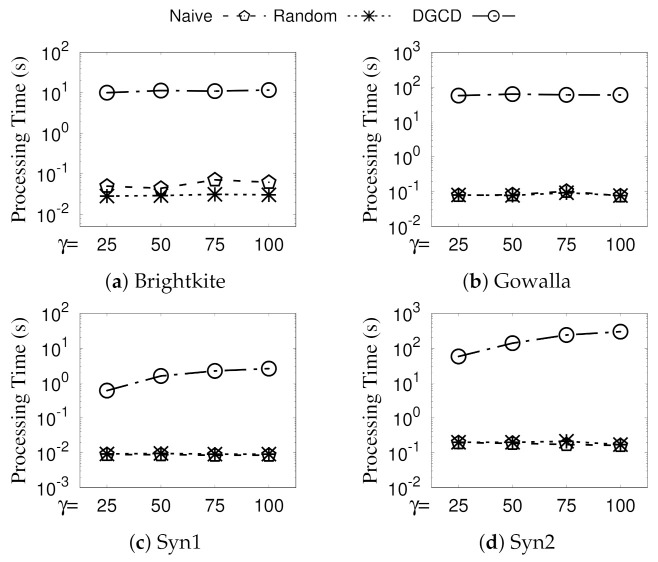
(Eval-V) Against existing algorithms (vary γ).

**Eval-V: Vary**γ. The experimental results of DGCD and our approaches by varying parameter γ are shown in Figure 9. The processing time of DGCD is kept steady because the structural similarity computations are not likely to be reduced under different physical distance thresholds γ. Regarding GSGD, as the value of γ changes, the run time is volatile. The reason for this is that, when γ increases, it is more likely that two vertices can be pruned by threshold γ; however, as the candidate communities become larger, computing MCC will take more time.

### 6.3. Performance of Top-*k* Densest GSG Mining

**Eval-VI: Total Number of Revisited GSGs.** As our algorithm Greedy needs to enumerate and keep all revisited GSGs $C^{A}$, we first evaluate the total number of revisited GSGs (i.e., $|C^{A}|$) to verify the feasibility of our strategy. Given μ=5, the results for different ϵ values are illustrated in Table 4. We can conclude that the size becomes larger when ϵ increases. Nevertheless, it is still manageable even for ϵ=0.4. Thus, our strategy of storing all revisited GSGs works in practice.

**Figure 10:**
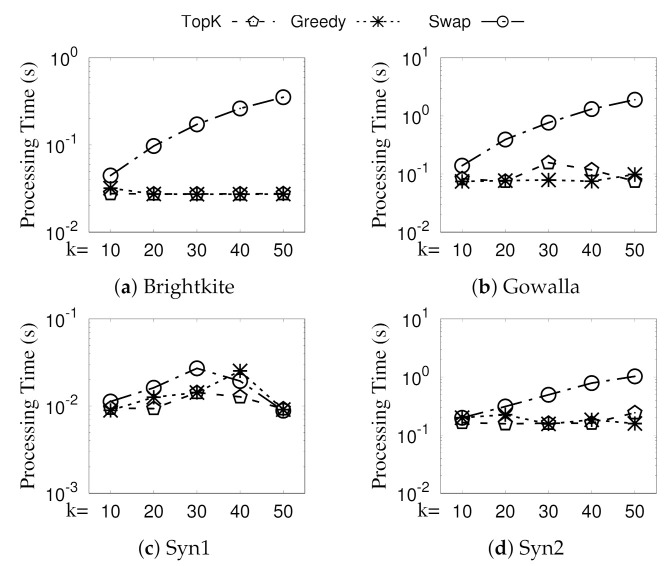
(Eval-VII) Efficiency evaluation (vary *k*).

**Figure 11:**
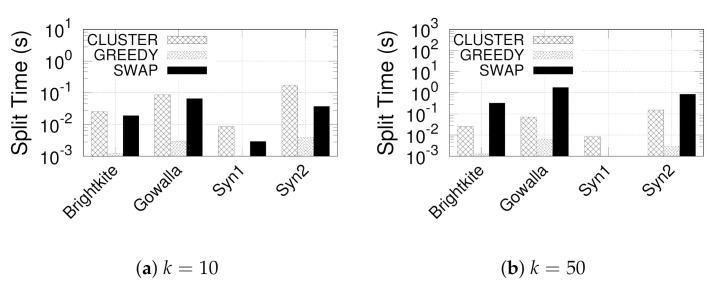
(Eval-VII) Split time of Greedy and Swap.

**Eval-VII: Vary *k*.**Figure 10 presents the processing time of Greedy, Swap, and TopK (we also implement a naive algorithm TopK, which directly chooses from $C^{A}$ the *k* GSGs with the largest individual densities) when varying *k*. Recall from Section 5 that all three of our algorithms consist of two phases: (Phase-I) generating all revisited GSGs $C^{A}$ and (Phase-II) selecting diversified top-*k* GSGs from $C^{A}$. We denote the second phase of Greedy, Swap, and TopK as Greedy, Swap, and TopK. The processing time of Greedy and TopK remain almost the same when *k* increases from 10 to 50 because Phase-I, which is irrelevant to *k*, is the dominating cost of Greedy and TopK. On the other hand, the run time of Swap increases significantly when *k* increases; this is because the time of Phase-II (streaming-like selection) in Algorithm 5, which increases with *k*, also becomes significant due to the improved Phase-I (see Figure 11). Nevertheless, as an approximation ratio of 1−1/e proven in Section 5, the performance of Swap is acceptable; note that Swap uses all of the optimization techniques of Greedy. Moreover, Greedy also runs faster than Swap when *k* becomes larger; this is due to the overhead of checking the condition of the swap for each newly generated GSG.

**Figure 12:**
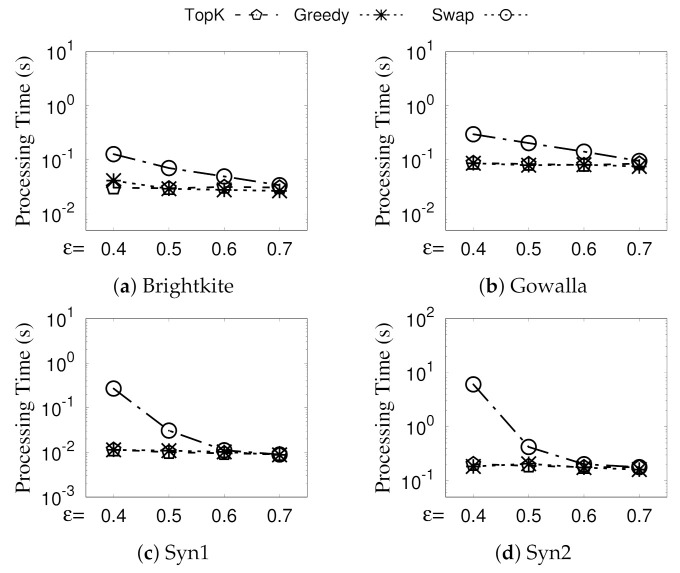
(Eval-VIII) Against existing algorithms (vary ϵ).

**Eval-VIII: Vary**ϵ. The processing time of Greedy, Swap, and TopK by varying ϵ is illustrated in Figure 12. In general, all three algorithms run faster for a larger ϵ; that is because the total number of revisited GSGs becomes smaller due to a high cohesive threshold ϵ, as shown in Table 4. Swap performs a little bit worse when ϵ is small, and Greedy and TopK are similar; however, the latter does not consider the overlap issue.

**Figure 13:**
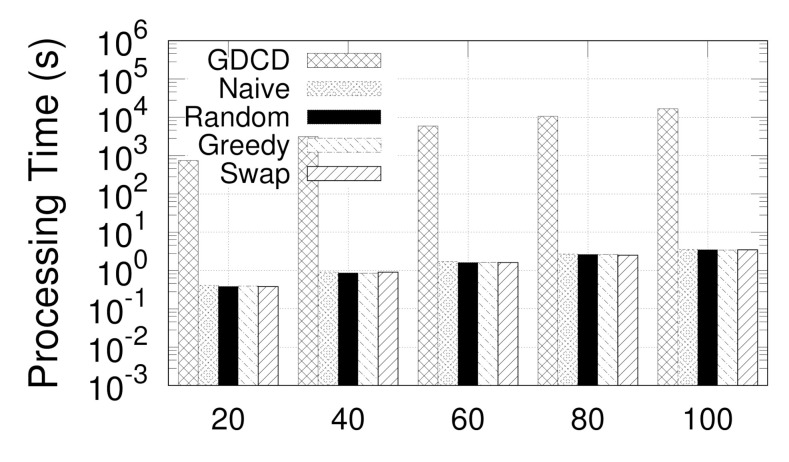
(Eval-IX) Scalability testing (vary |V|).

**Eval-IX: Scalability Testing.** In this experiment, we try to evaluate the scalability of our approaches on Syn3. For the graph, we randomly generate induced subgraphs with 20%, 40%, 60%, 80%, and 100% of the vertices of the original one. Given ϵ=0.4 and μ=5, the results are shown in Figure 13, where the *x*-axis shows the number of vertices in the subgraph. Generally, the run time of all algorithms increases along with the increasing number of vertices |V| due to the increase in the graph to be processed. Nevertheless, Greedy consistently outperforms Swap, and the improvement is up to two orders of magnitude. Thus, Greedy scales to large graphs as a result of our optimization techniques.

## 7. Conclusions

In this paper, we formulated a problem of detecting significant geo-social groups (GSGs) over a massive graph with location labels on vertices, where a GSG has not only a cohesive social but also spatial compactness. We proposed a new three-step paradigm and developed a highly efficient algorithm, GSGD, with several optimization techniques for enumerating all significant GSGs. Moreover, we extended the GSGs detection to top-*k* geo-social group (T*k*GSG) mining that identifies a set of important and diverse communities. We proved that our T*k*GSG mining problem is NP-hard and hard to approximate as well. Nevertheless, we proposed a greedy algorithm Greedy with an approximately ratio of 1−1/e and a streaming-like algorithm Swap. Experiments on large real and synthetic networks show that our approach outperforms the existing approaches by over one order of magnitude and demonstrates the effectiveness of our new GSG model. As a possible future direction, developing other cohesiveness measures in our model definition might be an interesting issue to be investigated.

## Figures and Tables

**Table 1:** The summary of notations.

Notation	Definition
G(V,E)	a graph with vertex set *V* and edge set *E*
n,m	the sizes of vertex and edge sets *V* and *E* resp.
G[C]	a subgraph of *G* induced by vertex set *C*
N[u]	the closed neighborhood [29] of vertex *u*
d[u]	the cardinality of N[u]
$G^{\prime }\subseteq G$	$G^{\prime }$ is a subgraph of *G*
$N_{\epsilon }\left[u\right]$	the ϵ-neighborhood of vertex *u*
σ(u,v)	the structural similarity between vertices *u* and *v*
MCC(C)	the minimum covering circle of vertex set *C*
TkGSG	Top-*k* Geo-Social Group

**Table 2:** Datasets used in our experiments (the last two columns are the average degree and the average clustering coefficient).

Type	Name	Vertices	Edges	$\hat{d}$	*c*
Real	Brightkite	58,228	214,078	3.68	0.17
	Gowalla	196,591	950,327	9.67	0.24
Synthetic	Syn1	10,000	97,750	19.55	0.2
	Syn2	100,000	980,295	19.61	0.2
	Syn3	1,000,000	9,778,000	19.56	0.2

**Table 3:** Total number of GSGs.

Dataset	Total Number of GSGs			
	ϵ=0.4	ϵ=0.5	ϵ=0.6	ϵ=0.7
Brightkite	467	261	105	49
Gowalla	1617	1064	537	234
Syn1	149	98	27	8
Syn2	1509	826	230	61

**Table 4:** Total number of revisited GSGs.

Dataset	Total Number of Revisited GSGs			
	ϵ=0.4	ϵ=0.5	ϵ=0.6	ϵ=0.7
Brightkite	636	330	133	60
Gowalla	1856	1166	575	244
Syn1	312	154	42	11
Syn2	3302	1433	372	84

## Data Availability

Not applicable.
